# Single cell RNA-sequencing delineates CD8^+^ tissue resident memory T cells maintaining rejection in liver transplantation

**DOI:** 10.7150/thno.96928

**Published:** 2024-08-12

**Authors:** Xinqiang Li, Shipeng Li, Yan Wang, Xin Zhou, Feng Wang, Imran Muhammad, Yurong Luo, Yandong Sun, Dan Liu, Bin Wu, Dahong Teng, Jinshan Wang, Kai Zhao, Qi Ling, Jinzhen Cai

**Affiliations:** 1 Organ Transplantation Center, Affiliated Hospital of Qingdao University, Qingdao, China.; 2 Institute of Organ Donation and Transplantation, Medical College of Qingdao University, Qingdao, China.; 3 Department of Hepatopancreaticobiliary Surgery, Henan Provincial People's Hospital, Zhengzhou University, Zhengzhou, China.; 4 First Hospital/First Clinical College of Shanxi Medical University, Taiyuan, China.; 5 Organ Transplant Center, Fujian Medical University Union Hospital, Fuzhou, China.; 6 Department of Surgery, The First Affiliated Hospital, Zhejiang University School of Medicine, Hangzhou, China.

**Keywords:** liver transplantation, tissue-resident memory T cells, multi-omics, graft rejection, immune tolerance

## Abstract

**Rationale:** Understanding the immune mechanisms associated with liver transplantation (LT), particularly the involvement of tissue-resident memory T cells (TRMs), represents a significant challenge.

**Methods:** This study employs a multi-omics approach to analyse liver transplant samples from both human (n = 17) and mouse (n = 16), utilizing single-cell RNA sequencing, bulk RNA sequencing, and immunological techniques.

**Results:** Our findings reveal a comprehensive T cell-centric landscape in LT across human and mouse species, involving 235,116 cells. Notably, we found a substantial increase in CD8^+^ TRMs within rejected grafts compared to stable ones. The elevated presence of CD8^+^ TRMs is characterised by a distinct expression profile, featuring upregulation of tissue-residency markers (CD69, CXCR6, CD49A and CD103^+/-^,), immune checkpoints (PD1, CTLA4, and TIGIT), cytotoxic markers (GZMB and IFNG) and proliferative markers (PCNA and TOP2A) during rejection. Furthermore, there is a high expression of transcription factors such as EOMES and RUNX3. Functional assays and analyses of cellular communication underscore the active role of CD8^+^ TRMs in interacting with other tissue-resident cells, particularly Kupffer cells, especially during rejection episodes.

**Conclusions:** These insights into the distinctive activation and interaction patterns of CD8^+^ TRMs suggest their potential utility as biomarkers for graft rejection, paving the way for novel therapeutic strategies aimed at enhancing graft tolerance and improving overall transplant outcomes.

## Introduction

The enduring success of liver transplantation (LT) is intricately linked to the intricate dynamics within the transplant immune microenvironment. Post LT, the orchestration of interactions between donor-derived cells and the recipient's immune system is pivotal for graft acceptance 1, 2. Allograft immunity represents a multifaceted process, arising from intricate interactions among various immune cell types, including lymphocytes, monocytes, macrophages, and dendritic cells. In this dynamic scenario, recipient alloreactive T cells identify donor alloantigens, presented either by donor-derived or recipient antigen-presenting cells. This recognition triggers an adaptive inflammatory immune response, a critical pathway leading to allograft rejection 3. T cells, composed of diverse subgroups, play a pivotal role in modulating the immune response, particularly in enhancing anti-donor reactions 4, 5. These subgroups include subsets such as CD4^+^ regulatory T cells (Tregs), related to immune tolerance, and cytotoxic CD8^+^ T cells, often associated with transplant rejection 6. Understanding the distinct functions of these T cell subsets with different locations is essential in transplant immunology. Therapeutic strategies targeting these cells are gaining momentum in transplant medicine. The quest to identify cellular markers and T cell subsets driving this rejection is vital for devising targeted monitoring strategies and therapeutic interventions, marking a substantial leap forward in transplant immune.

Tissue-resident memory T cells (TRMs), a specialized subset of the immune arsenal, have emerged as pivotal mediators in the immunology of nonlymphoid tissues 7, 8 and solid organ transplantation, influencing the fate of transplanted hearts 9, kidneys 10, and lungs 11, frequently tipping the balance toward rejection. TRMs are distinct both phenotypically and transcriptionally from circulating memory T cells. They are characterized by the expression of activation, retention, and adhesion markers such as C-type lectin CD69, integrin CD103, and are capable of producing effector cytokines like interferon-γ (IFN-γ), granzyme B (GZMB), and proliferating locally 12, 13. Both CD4^+^ TRMs and CD8^+^ TRMs have been identified as playing pivotal, yet differing, roles during the course of organ transplantation. Notably, long-lived alloreactive CD4^+^ TRMs, residing in organs or tissues other than the transplant site, have been shown to significantly contribute to organ allograft rejection, as evidenced in skin and heart transplantation studies 9. Further research has demonstrated that antigens and IL-15, presented by dendritic cells, are crucial for the maintenance of CD8^+^ TRMs in scenarios as observed in a mouse kidney transplantation model 14. The liver, recognized for its immunotolerant environment, presents a unique set of transplantation challenges distinct from these organs 15. Liver-resident macrophages, named Kupffer cells, reside in the transplanted liver and contribute to the maintenance of transplant immune. While our previous studies have mapped the single-cell environment of the transplanted liver, highlighting the significant roles played by T cell and myeloid cell populations 16, there remains a notable gap in our understanding of the specific contributions of TRMs to liver transplant outcomes.

Our current investigation seeks to bridge this gap by dissecting the phenotypic properties and critical functions of TRMs within LT contexts, employing a comprehensive multi-omics approach that spans single-cell RNA sequencing, bulk RNA sequencing, and benchmark experimental assays. This study is poised to shed light on the complex roles of TRMs, delving into their potential to trigger, sustain, or regulate immune responses post-transplantation. By elucidating these roles, we aim to deepen our understanding of the intricate equilibrium between immune-mediated rejection and tolerance of LT.

## Results

### Construction of the Single-Cell Landscape of Human Transplanted Liver

**Figure 1:**
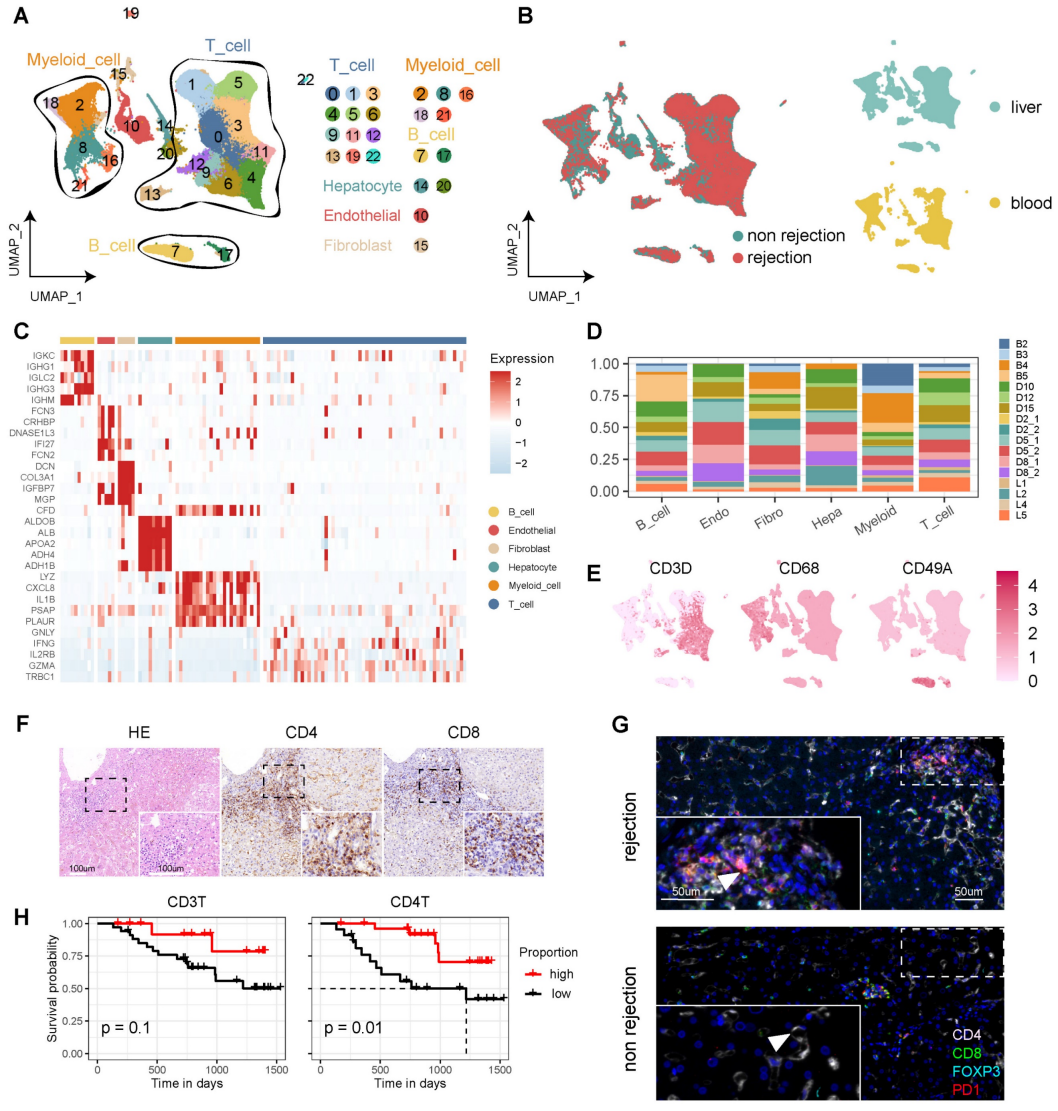
** Single-Cell Atlas of Human Liver Transplantation.** A) UMAP plot depicting cell type identification of 115,026 high-quality cells in human liver transplantation. B) UMAP plots colored by spatial distribution of cells among diagnoses (rejection and non-rejection) and tissues (liver and blood). C) Heatmap displaying the top 5 genes of each cell type. D) Bar plots illustrating the proportion of cell types in each sample. E) UMAP plots showing the expression of CD3D, CD68, and CD49A across the landscape. F) HE and immunohistochemistry of CD4 and CD8 in rejection-transplanted liver. Insets highlight areas of T cell infiltration. Whole image scale bar, 200 μm. Inset scale bar, 100 μm. G) Multi-immunohistochemistry of rejection and non-rejection transplanted liver for DAPI (blue), CD4 (white), CD8 (green), FOXP3 (cyan), and PD1 (red). White arrows point to cells co-expressing CD8 and PD1. Whole image scale bar, 200 μm. Inset scale bar, 50 μm. H) KM analysis showing proportion of CD3^+^ and CD4^+^ T cells in the peripheral blood of LT patients for one month with prognosis.

To delineate the cellular dynamics of human LT, we established a comprehensive single-cell landscape from 17 samples, encompassing both liver (n = 13) and blood (n = 4), including cases with (n = 9) and without rejection (n = 8) (Figure S1A, Table S1). To enhance data analysis and minimize batch effects, we integrated samples from both DNBelab C4 and 10X Genomics sequencing platforms. This landscape comprises 115,026 high-quality cells, categorized into 23 clusters post-filtration, and annotated with canonical markers. These markers include CD3D, CD3E, and CD3G for T cells (69.99%); CD68, CD14, and CD163 for myeloid cells (15.78%); COL1A2, COL3A1, and ACTA2 for fibroblasts (1.25%); ERG, ENG, and CD34 for endothelial cells (4.51%); ALB, TF, and TTR for hepatocytes (1.86%) (Figure 1A, C, and Table S1). The analysis revealed a distinct distribution of diagnostic categories and tissue types across the cellular landscape (Figure 1B). Notably, T cells and myeloid cells constituted the majority of this landscape, displaying variability across the samples—particularly the T cells (Figure 1D-E, Figure S1B).

Immunohistochemistry (IHC) analyses indicated heightened expression levels of CD4 and CD8 in rejection samples (Figure 1F). Multi-immunohistochemistry (mIHC) further highlighted that CD8^+^PD1^+^ T cells were prevalent in samples from rejection cases (Figure 1G and Figure S1C). Furthermore, we conducted statistical analyses to examine circulating T cells and their correlation with patient outcomes following transplantation. Our flow cytometry analysis revealed that a higher proportion of CD3^+^ and CD4^+^ T cells in the peripheral blood of LT patients for one month is associated with a more favorable prognosis (Figure 1H).

### CD4^+^ TRMs Exhibit Dual Phenotypic Roles in Liver Transplantation

**Figure 2:**
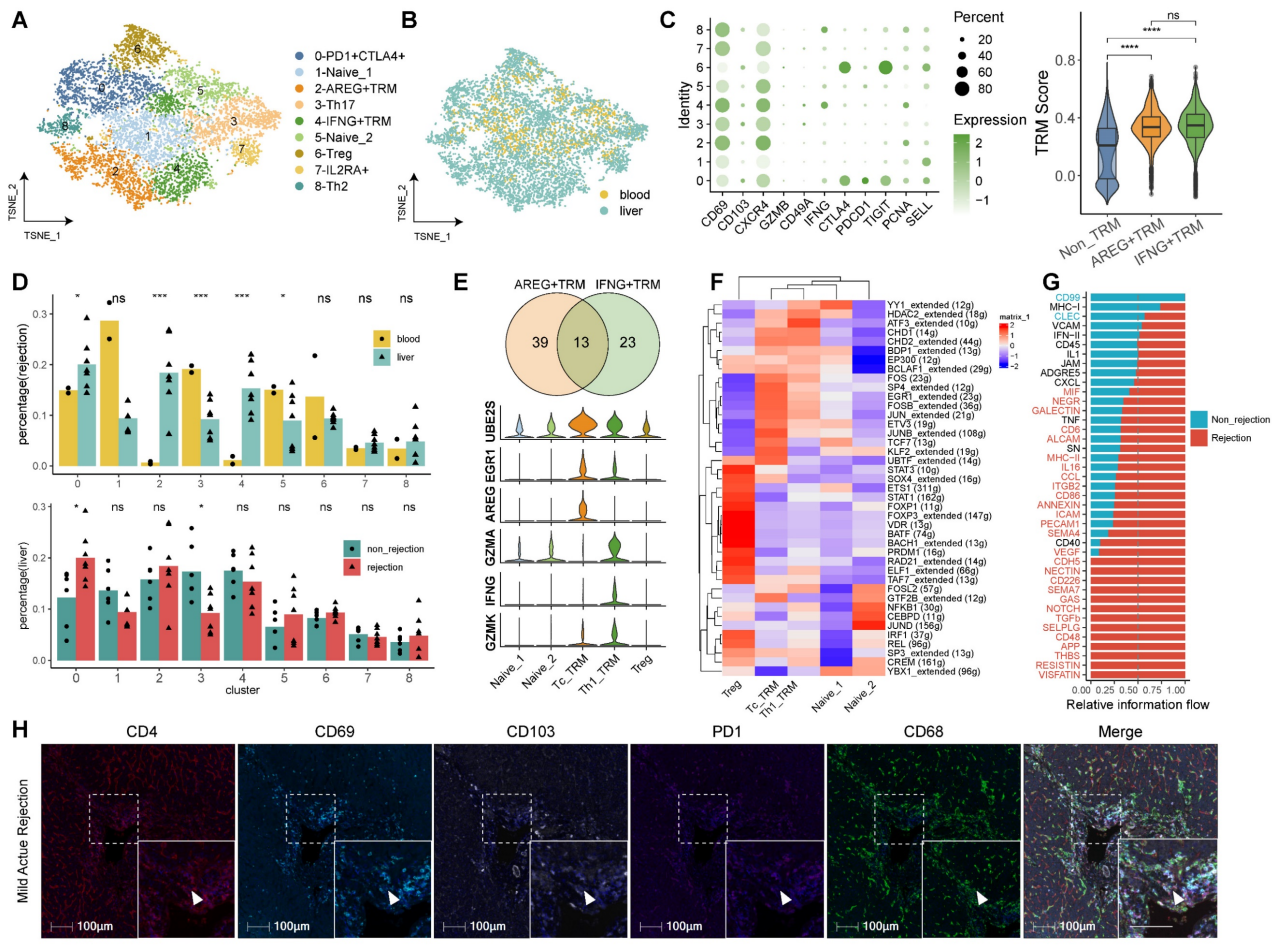
** Identifying CD4^+^ T Cell Subsets in Human Transplanted Liver and Blood.** A) tSNE plot for re-clustering and cell type identification of 8,836 CD4^+^ T cells. B) tSNE plot colored by spatial distribution of cells among tissues (liver and blood). C) Dot plots showing the expression of TRM-related genes among clusters. Violin plots illustrating the TRM score among AREG^+^ TRM, IFNG^+^ TRM, and other CD4^+^ T subsets. D) Fractions of CD4^+^ T subsets among tissues (liver and blood) in rejection samples and diagnoses (rejection and non-rejection) in liver samples. E) Venn plot and violin plots showing the common and different marker genes between two CD4^+^ TRMs. F) Heatmap of the t-value for the area under the curve score of expression regulation by transcription factors, as estimated using SCENIC. G) Bar plots illustrating the cellular communication between CD4^+^ TRMs and other cells. Red pathways indicate rejection, while blue pathways indicate non-rejection in prevalence. H) Multi-immunohistochemistry of rejection-transplanted liver for DAPI (blue), CD4 (red), CD69 (cyan), CD103 (white), PD1 (purple), and CD68 (green). White arrows point to cells co-expressing CD4, CD69, and CD103. Whole image/insert scale bar, 100 μm.

Our exploration of CD4^+^ TRMs highlights their potential to modulate immune responses in LT. We identified nine CD4^+^ T cell subclusters, including Naïve T, Treg, Th2, Th17, and TRM cells, each exhibiting unique tissue distributions (Figure 2A-B). The predominant cluster, characterized by the upregulation of immune checkpoints (PD1, CTLA4, TIGIT), was designated as PD1^+^CTLA4^+^ T cells. Based on gene expression profiles, we identified two distinct phenotypes of CD4^+^ TRMs: C2-AREG^+^ TRM and C4-IFNG^+^ TRM. Both expressed the markers CD69, CXCR4, and PCNA, typically associated with CD4^+^ TRMs, while C4-IFNG^+^ TRM showed higher expression of IFNG, indicating a TRM1 phenotype. TRM scoring across subclusters affirmed that both C2-AREG^+^ TRM and C4-IFNG^+^ TRM phenotypes possessed significantly elevated TRM scores compared to others, though no notable differences were observed between the two (Figure 2C).

The CD4^+^ subset showed different distribution among tissues and diagnoses. In rejection status, C0-PD1^+^CTLA4^+^ T, C2-AREG^+^ TRM and C4-IFNG^+^ TRM were predominantly found in liver, while C3-Th17 and Naïve T (C1-Naive_1 and C5-Naive_2) showed high proportion in blood. In liver tissue, C0-PD1^+^CTLA4^+^ T showed high proportion in rejection samples while C3-Th17 was downregulated in rejection. However, no significant differences were detected between C2-AREG^+^ TRM and C4-IFNG^+^ TRM when comparing rejection to non-rejection liver tissues (Figure 2D).

Differential gene expression analysis between the two CD4^+^ TRM clusters identified 52 and 36 uniquely expressed genes for C2-AREG^+^ TRM and C4-IFNG^+^ TRM, respectively, with a commonality of 13 signature genes, including transcription factors FOSB, FOS, JUN, and NR4A1, as corroborated by transcription factor analysis. Compared with CD4^+^ Treg and Naïve T cells, distinct expression patterns were noted, where C2-AREG^+^ TRM upregulated UBE2S, AREG, and RGR1, while C4-IFNG^+^ TRM showed heightened levels of GZMA, IFNG, GZMK, STAT1, STAT4, and CXCR3, indicative of a TRM1 phenotype (Figure 2E-F, Figure S2A and Table S2).

Functional enrichment analysis was performed to distinguish the difference between the two clusters (Table S2). Gene Ontology (GO) pathway analysis revealed overlapping pathways for both CD4^+^ TRM clusters in myeloid cell differentiation, negative regulation of protein phosphorylation, and activation of the stress-activated MAPK cascade. Unique to C2-AREG^+^ TRM were pathways involving epithelial cell migration, regulation of epithelial cell proliferation, and response to fibroblast growth factor. Conversely, C4-IFNG^+^ TRM was associated with the extrinsic apoptotic signaling pathway, positive regulation of hepatocyte proliferation, and modulation of steroid metabolic processes, alongside enhancement of cytokine and interleukin-1 production (Figure S2B-C, Table S2).

Cellular communication analysis indicated that pathways in rejected tissues predominantly involved TGFβ, NOTCH, GAS, and NECTIN signaling, whereas both rejection and non-rejection samples shared pathways related to IFN-II, CXCL, and TNF signaling (Figure 2G). To validate the role of CD4^+^ TRM in human LT, we conducted mIHC on LT samples exhibiting rejection. Our analysis revealed that CD4^+^ TRM, characterized by high expression of CD69 and CD103 and low expression of PD-1, were actively involved in various stages of rejection, including mild acute, severe acute, and chronic rejection. Additionally, these cells were observed to interact significantly with myeloid cells (Figure 2H, Figure S2D).

### CD8^+^ TRMs Predominate in Rejected Transplanted Liver Tissues

**Figure 3:**
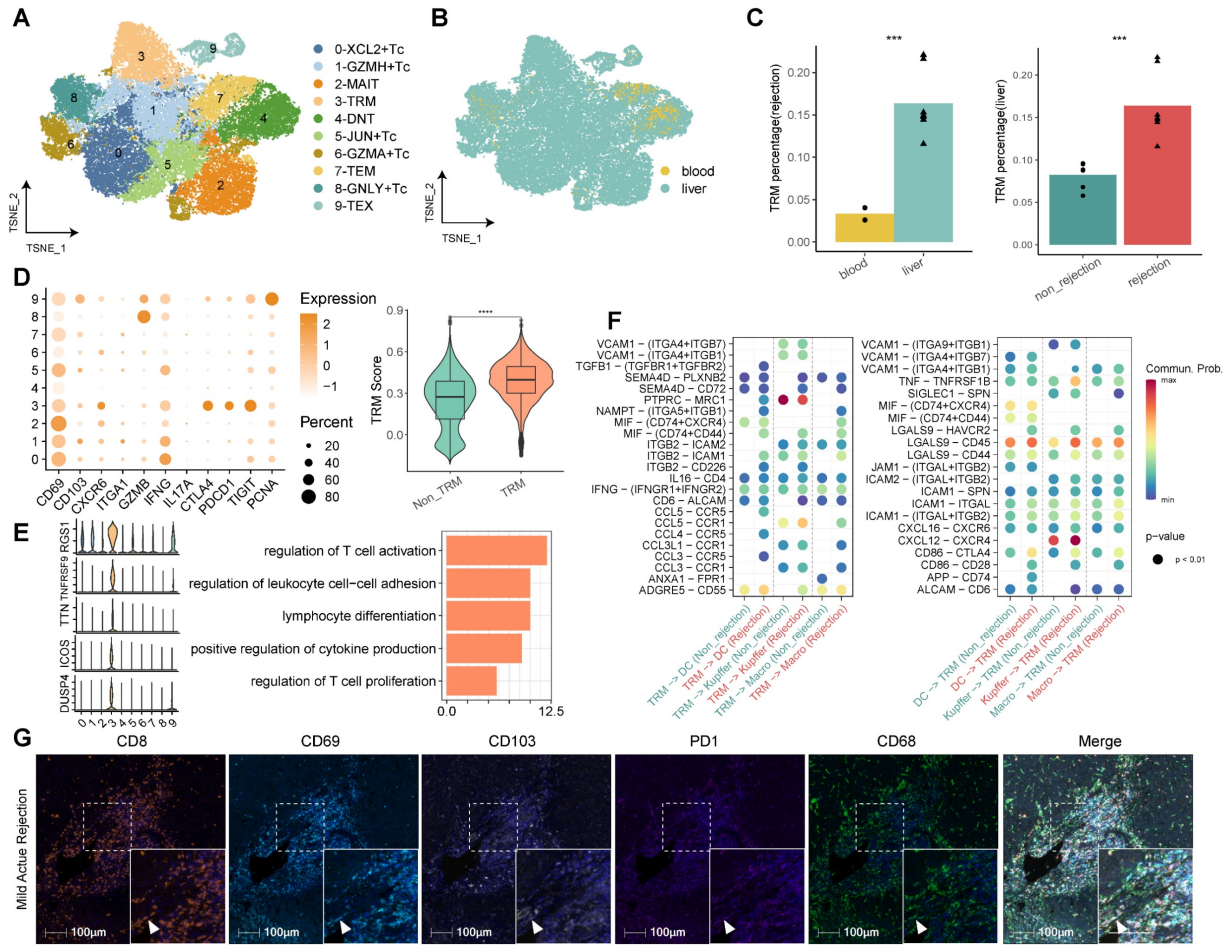
** Identifying CD8^+^ T Cell Subsets in Human Transplanted Liver and Blood.** A) tSNE plot for re-clustering and cell type identification of 46,117 CD8^+^ T cells. B) tSNE plot colored by spatial distribution of cells among tissues (liver and blood). C) Fractions of CD8^+^ TRMs among tissues (liver and blood) in rejection samples and diagnoses (rejection and non-rejection) in liver samples. D) Dot plots showing the expression of TRM-related genes among clusters. Violin plots illustrating the TRM score between CD8^+^ TRMs and other CD8^+^ T subsets. E) Violin plots showing the top DEGs of CD8^+^ TRMs. Bar plots illustrating the GO pathway enrichment analysis for DEGs of CD8^+^ TRMs. F) Dot plots showing the cellular communications between CD8^+^ TRMs and Myeloid cell subsets between non-rejection (green) and rejection (red) samples. G) Multi-immunohistochemistry of rejection-transplanted liver for DAPI (blue), CD8 (orange), CD69 (cyan), CD103 (white), PD1 (purple), and CD68 (green). White arrows point to cells co-expressing CD8, CD69, CD103, and PD1. Whole image/insert scale bar, 100 μm.

Our comprehensive analysis of CD8^+^ T cells, facilitated by re-clustering within the established single-cell landscape, delineated 10 discrete clusters. We annotated these clusters according to their functional attributes, identifying cytotoxic T cells (Tc), tissue-resident memory T cells (TRM), effector memory T cells (TEM), exhausted T cells (TEX), mucosal-associated invariant T cells (MAIT), and double negative T cells (DNT) (Figure 3A, Table S3). Among the cytotoxic cohorts (Tc), five clusters were characterized—C0-XCL2^+^ Tc, C1-GZMH^+^ Tc, C5-JUN^+^ Tc, C6-GZMA^+^ Tc, and C8-GNLY^+^ Tc—all expressing classic cytotoxicity-associated markers such as GZMA, GZMH, and NKG7 (Table S3). Notably, each Tc subset exhibited distinct cytokine profiles, with C0-XCL2^+^ Tc marked by elevated cytokines (XCL2, XCL1, CCL4, CCL3, IFNG), C5-JUN^+^ Tc by transcriptional regulators (JUN, FOSB, FOS, GADD45B, EGR1), and C8-GNLY^+^ Tc by effector molecules (GNLY, FGFBP2, CX3CR1).

The tissue distribution analysis of CD8^+^ T cell subclusters revealed a liver dominance, with significant enrichment in rejected transplants (Figure 3B). Cluster 3, identified as CD8^+^ TRM, was particularly overrepresented in rejected liver tissues, characterized by an upregulation of tissue residency (CD69, CD103, CXCR6 and CD49A), cytotoxic (GZMB, IFNG), immune checkpoint (PD1, CTLA4, TIGIT) and proliferative (PCNA) markers. To corroborate the TRM designation, we employed TRM scoring, confirming that C3-TRM displayed substantially higher scores in comparison to other clusters, denoting its specificity (Figure 3C-D).

The top genes of CD8^+^ TRMs include RGS1, TNFRSF9, TTN, ICOS, and DUSP4, which were partly reported to function with TRMs 17-19. Functional pathway analysis revealed that differentially expressed genes (DEGs) within CD8^+^ TRMs were significantly involved in pathways germane to T cell activation, proliferation, and differentiation, cytokine production, antigen processing and presentation, and notably, allograft rejection (Figure 3E, Figure S3). Interrogation of cellular communication networks elucidated that TRMs engaged in extensive dialogue with myeloid cells, including dendritic cells, Kupffer cells, and monocyte-derived macrophages. In the context of rejection, TRMs exhibited an augmented secretion profile of cytokines (TGFB1, IFNG, CCL5, CCL4, CCL3L1) and demonstrated enhanced reception of chemokines (CXCL12, CXCL16) from myeloid cells (Figure 3F). Furthermore, utilizing mIHC, we investigated the presence of CD8^+^CD103^+/-^ TRMs in LT samples undergoing different stages of rejection, encompassing mild acute, severe acute, and chronic rejection. These TRMs were identified by their high expression levels of CD69, CD103, and PD-1. Our analysis also highlighted their interactions with myeloid cells within these various rejection contexts (Figure 3G, Figure S3B).

### CD8^+^ TRM Marker Expression Distinguishes Rejection in Liver Transplantation

**Figure 4:**
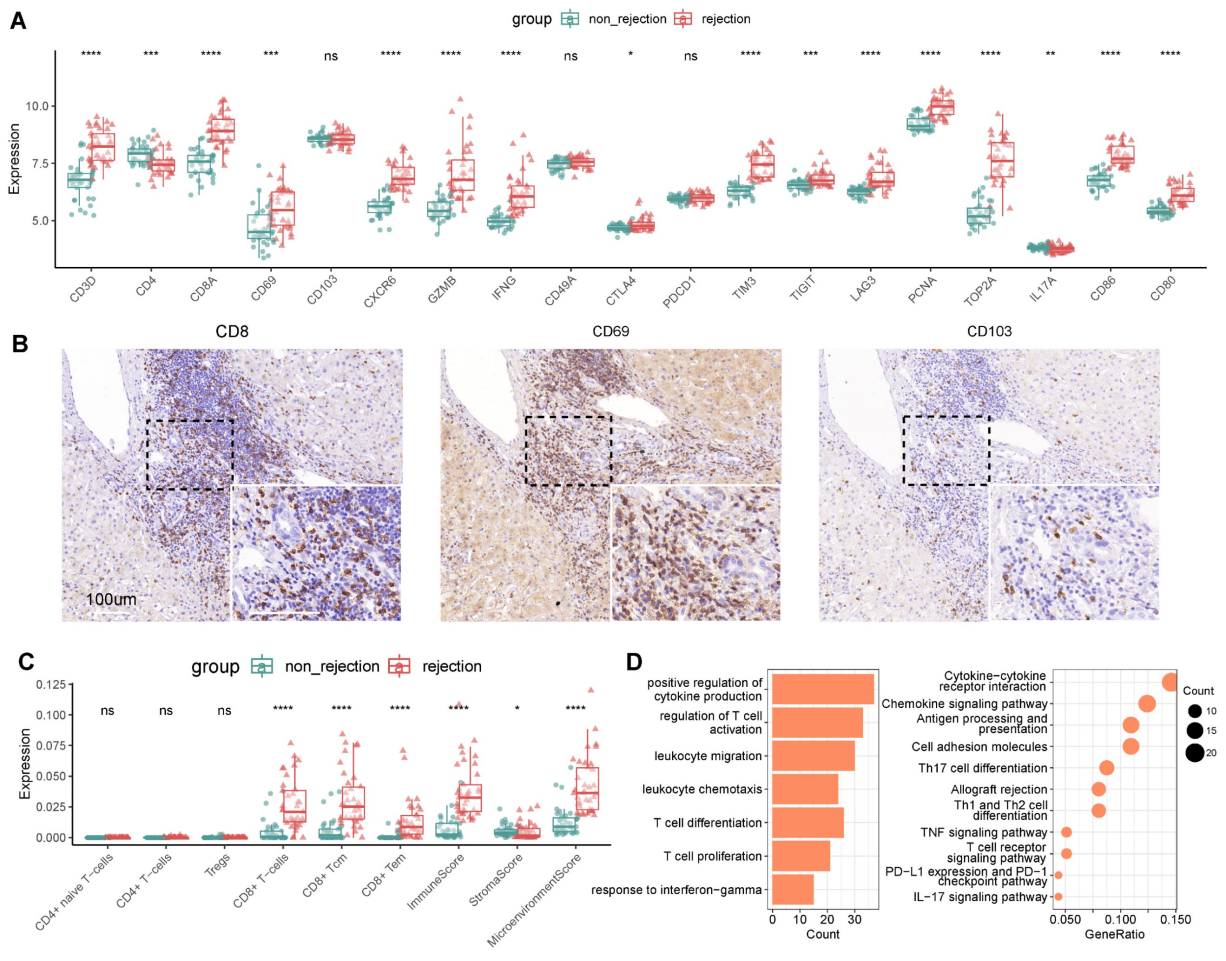
** CD8^+^ TRM Marker Expression Distinguishes Rejection in Liver Transplantation.** A) Gene expression of TRM-related markers between non-rejection (n = 37) and rejection (n = 37) samples using bulk RNA-seq. B) Immunohistochemistry of CD8, CD69, and CD103 in rejection-transplanted liver. Whole/insert image scale bar, 100 μm. C) Analysis of immune infiltration between non-rejection and rejection transplanted liver. D) Bar plots and dot plots illustrating the GO and KEGG pathway enrichment analysis for DEGs between non-rejection and rejection tissues.

In investigating the potential of TRMs as biomarkers for LT outcomes, we utilized bulk RNA sequencing on an independent cohort to discern the expression patterns associated with transplant rejection. The cohort included multiple liver biopsy samples from cases both with (n = 37) and without (n = 37) rejection. Differential expression analysis indicated that a majority of CD8^+^ TRM markers were upregulated in rejection cases. This included markers for CD8^+^ T cells (CD3D, CD8), tissue residency (CD69, CXCR6), immune checkpoints (CTLA4, TIM3, TIGIT, LAG3), cytotoxic (IFNG, GZMB), and proliferation (PCNA, TOP2A), along with costimulatory molecules CD86 and CD80 (Figure 4A), which were validated by another independent cohort (n = 7) (Figure S4). These findings were in agreement with our single-cell analyses, confirming the prevalence of CD8^+^ TRMs in rejected graft tissues (Figure 3).

Contrastingly, certain markers such as CD103, CD49A, and PD1 did not exhibit significant differential expression between rejected and non-rejected samples. Concurrently, markers indicative of CD4^+^ Th17 cells, specifically CD4 and IL17A, were significantly downregulated in rejected samples, corroborating our earlier observations (Figure 2D). Immunohistochemical (IHC) validation suggested an inclination of CD8, CD69, and CD103 expressions towards rejected samples (Figure 4B).

Using a predictive model for immune infiltration in post-transplant tissues, we observed that tissues from rejected transplants harbored significantly higher proportions of CD8^+^ T cells and CD8^+^ memory T cells. They also demonstrated elevated immune and microenvironmental scores compared to non-rejected tissues. However, populations of CD4^+^ T cells, naïve CD4^+^ T cells, and Treg cells displayed no significant variance between rejected and non-rejected samples (Figure 4C), aligning with our previous single-cell analyses (Figure 2A-B, Figure 3A-B).

Further differential expression analysis of genes associated with rejection revealed an upregulation of T cell-mediated pathways, encompassing the regulation of T cell activation, proliferation, and differentiation. Cytokine-mediated pathways were also prominent, particularly those governing the positive regulation of cytokine production and the response to interferon-gamma (Figure 4D, Table S4).

### Dynamic Shifts of CD8^+^ TRMs in Mouse Liver Transplantation Models

**Figure 5:**
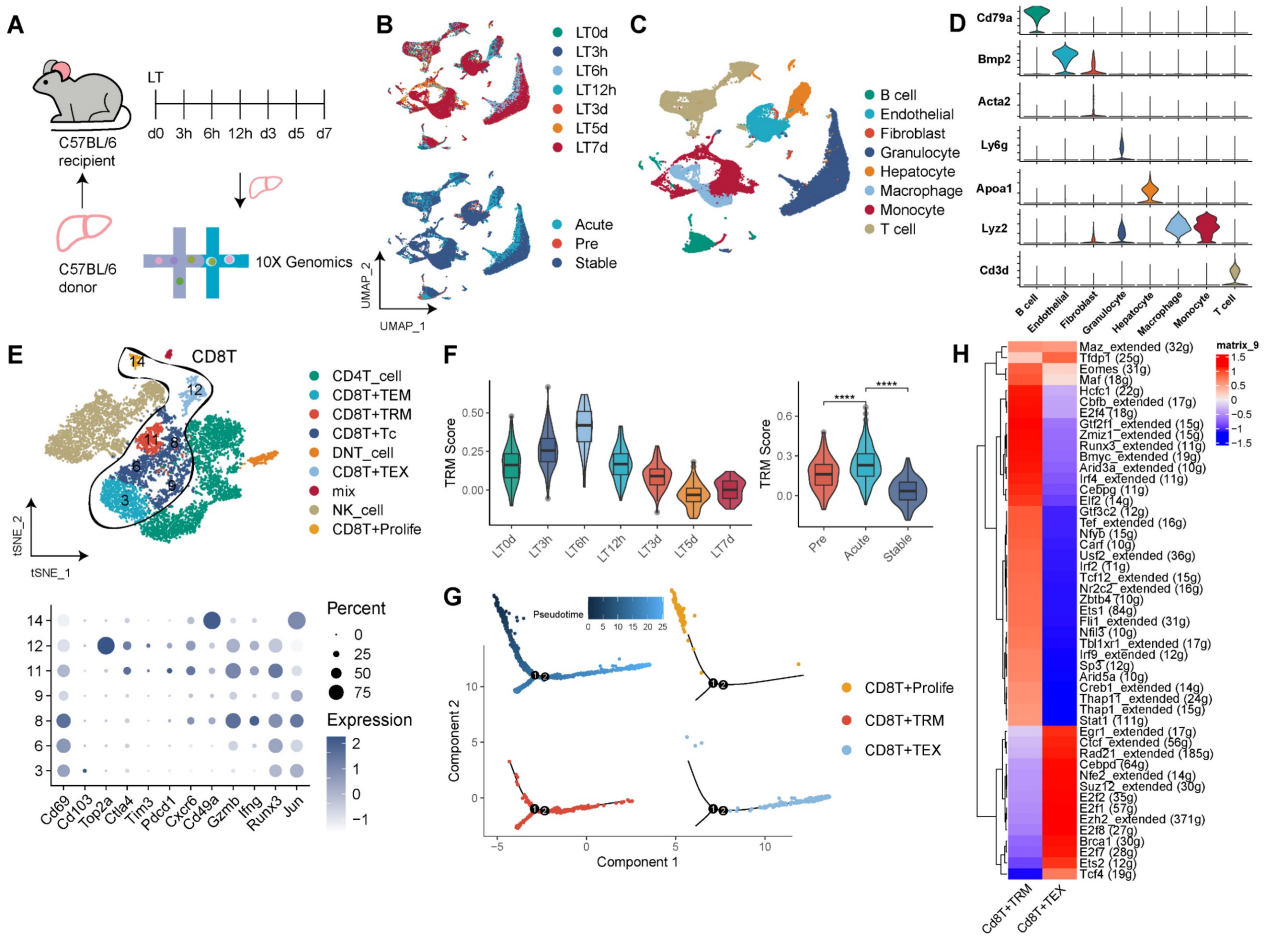
** CD8^+^ TRMs Showing Dynamic Timeline Phenotypes in Mouse Liver Transplantation Models.** A) C57BL/6 liver allografts were transplanted into C57BL/6 recipients, and single-cell RNA-seq analysis was performed using 10X genomics. B) UMAP plots showing the distribution of cells among dynamic timelines (pre-LT, 3h, 6h, 12h, 5d, 7d post-LT) and phases (pre, acute, and stable). C) Single-cell atlas of mouse LT in dynamic timelines, comprising different immune and stromal cells. D) Expression of canonical cell markers including Cd3d, Lyz2, Apoa1, Ly6g, Acta2, Bmp2, and Cd79a. E) tSNE plots for re-clustering and cell type identification of 11,480 T cells, especially CD8^+^ T cells. Dot plots showing the expression of TRM-related genes among CD8^+^ T clusters. F) Violin plots showing the TRM score among dynamic timelines and phases. G) Pseudotime analysis with identified CD8^+^ proliferative T cells, TRM, and TEX cells. H) Heatmap of the t-value for the area under the curve score of expression regulation by transcription factors between CD8^+^ TRM and TEX cells.

Our observations in human LT underscore the pivotal role of TRMs, particularly CD8^+^ TRMs. To expand upon these insights, we explored the CD8^+^ TRM profile within a mouse model of LT (Figure 5A). Data were gathered from seven time points across the LT timeline (pre-LT, 3h, 6h, 12h, 3d, 5d, 7d post-LT), corresponding to the pre-transplant (LT0D), acute (LT3h, LT6h, LT12h), and stable (LT3d, LT5d, LT7d) phases of the mouse LT model in a tolerance setting. Our analysis yielded 30 distinct clusters (Figure 5B, Figure S5A-B), and we constructed a comprehensive cellular atlas of mouse LT, comprising 63,049 cells, marked by canonical cell-type identifiers such as Cd3d for T cells, Cd79a for B cells, Lyz2 for monocytes, Adgre1 for macrophages, Ly6g for granulocytes, Bmp2 for endothelial cells, Acta2 for fibroblasts, and Apoa1 for hepatocytes (Figure 5C-D, Figure S5C).

A refined clustering of T cells unveiled subsets including CD8^+^ Tc, CD8^+^ TRM, CD8^+^ TEM, CD8^+^ TEX, CD8^+^ Proliferative T cells, CD4^+^ T cells, and DNT cells. The CD8^+^ TRM cluster (c11) notably expressed high levels of Cd69, Cxcr6, Gzmb, Ifng, Runx3, and Jun, and low levels of Cd103 (Figure 5E, Figure S5C). Temporal TRM scoring revealed dynamic fluctuations, with the acute phase of mouse LT displaying significantly elevated TRM scores compared to both pre-transplant and stable phases (Figure 5F). Pseudotime trajectory analysis suggested that CD8^+^ TRMs occupy a middle-to-late stage, akin to CD8^+^ TEX, whereas the CD8^+^ Proliferative subset was positioned in the early phase, indicating a transition of effector CD8^+^ T cells to functional TRMs, which might sustain localized rejection in the transplanted liver (Figure 5G).

Transcription factor profiling of CD8^+^ TRMs in the mouse model unveiled an abundance of transcripts, including Eomes, Runx3, Maf, Irf4, and Stat1 (Figure 5H). Investigating cellular communication, we mapped the interplay between TRMs and other tissue-resident cells including Kupffer cells, endothelial cells, and fibroblasts across time (Figure S5E-F). Significantly, our analysis of cellular communications revealed variations in the number and intensity of signals between TRMs and Kupffer cells across different phases of LT. In the acute phase, there was an increased prevalence of signals, notably Pd-l1/Pd-1 interactions and CC chemokines, such as Ccl3, Ccl4, and Ccl5, predominantly originating from Kupffer cells. Conversely, in the stable phase, a higher incidence of signals involving Cxcl chemokines, including Cxcl4, Cxcl13, and Cxcl16, was observed (Figure S5F). Complementing these findings, bulk RNA sequencing data revealed that MHC-mismatched mouse LT models exhibited higher expression of TRM-related genes compared to MHC-matched counterparts, demonstrating notable dynamic changes post-transplantation (Figure S5G).

### Single-cell Transcriptomics and Cellular Communication analysis of CD103^-^CD8^+^ TRMs in the Rejection and Tolerance Phases of Mouse Liver Transplantation

**Figure 6:**
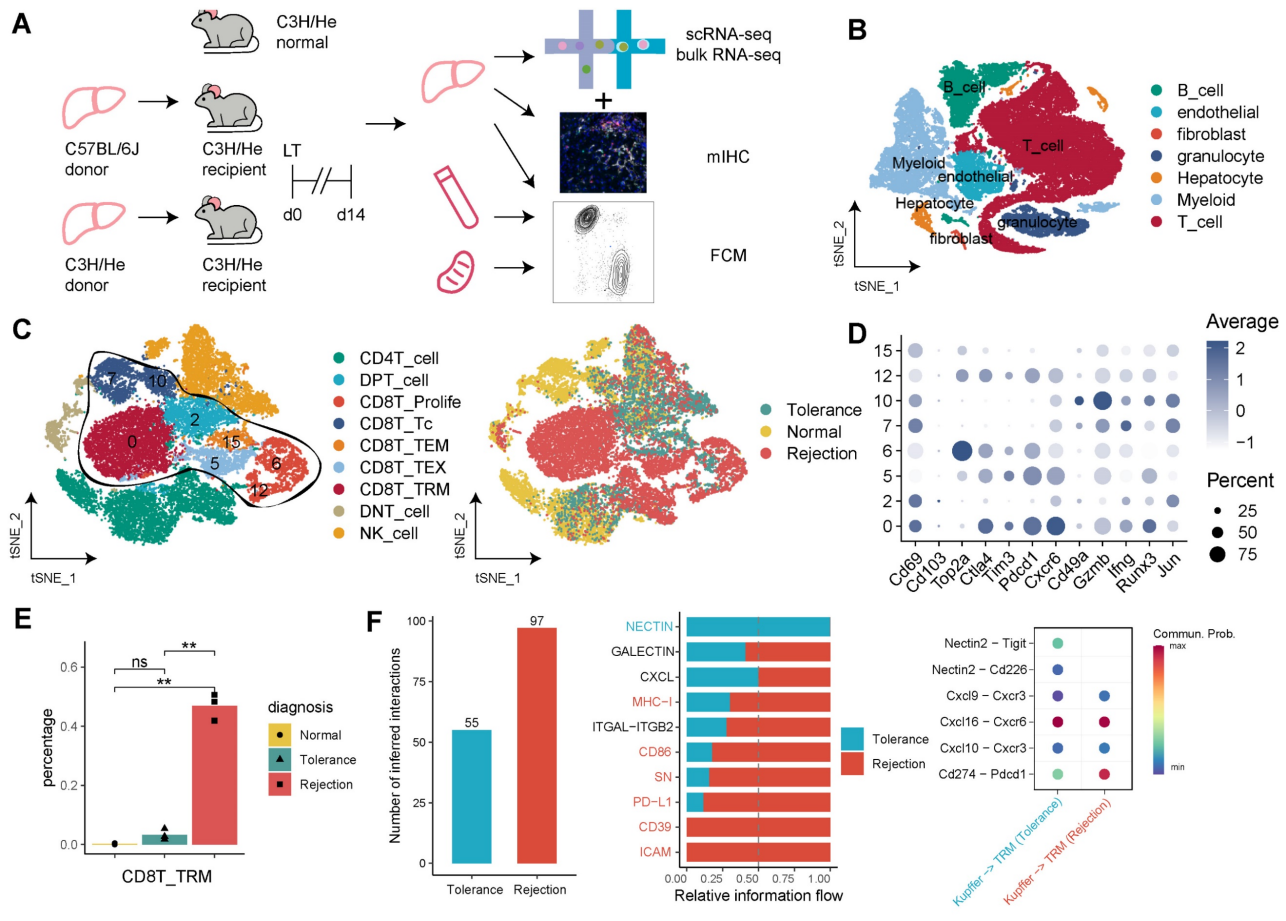
** Single-Cell Omics Analysis of CD8^+^ TRMs in Rejection and Tolerance Phases of Mouse Liver Transplantation.** A) C57BL/6J (n = 3) or C3H/He (n = 3) liver allografts were transplanted into C57BL/6 recipients, and single-cell RNA-seq analysis was performed using 10X genomics. B) Single-cell atlas of mouse LT in different diagnoses, comprising different immune and stromal cells. C) tSNE plots for re-clustering, cell type identification, and tissue distribution of 28,408 T cells, especially CD8^+^ T cells. D) Dot plots showing the expression of TRM-related genes among CD8^+^ T clusters. E) Fractions of CD8^+^ TRMs among diagnoses (rejection, non-rejection, and normal). F) Bar plots and dot plots illustrating the cellular communication between CD8^+^ TRMs and Kupffer cells. Red indicating rejection and cyan indicating non-rejection.

To elucidate the roles of CD8^+^ TRMs across different immunological outcomes in a mouse model of LT, we initially performed scRNA-seq on samples from mice undergoing LT with rejection (C57BL/6 to C3H/He; n = 3), tolerance (C3H/He to C3H/He; n = 3), and from normal, healthy controls (C3H/He; n = 3) and constructed a immune landscape of 57,041 cells, including T cells, B cells, myeloid cells, granulocytes, hepatocytes, fibroblasts, and endothelial cells, for mouse LT (Figure 6A, B). We focused on the T cell compartment, comprising 28,408 cells, and annotated subpopulations including CD8^+^ Tc, CD8^+^ TRM, CD8^+^ TEM, CD8^+^ Proliferative T cells, CD4^+^ T cells, and DNT cells (Figure 6C). Using a panel of established markers (Cd69, Pd1, Cxcr6, Gzmb, Ifng, Runx3, Jun), we identified CD8^+^ TRMs, which showed low expression of Cd103 and were significantly more abundant in rejection samples compared to those from tolerance and normal states (Figure 6D-E).

Cellular communication analysis in the rejection state highlighted an intensified signaling activity of TRMs. This included enhanced interactions via MHC-1, CD86, SN, PD-L1, CD39, and ICAM pathways. Notably, Kupffer cells in the rejection group exhibited robust signaling with CD8^+^ TRMs, particularly through Cxcl16-Cxcr6, Cxcl10-Cxcr3, and Pdl1-Pd1 pathways (Figure 6F, Figure S6A). Additionally, CD8^+^ TRMs in rejection samples demonstrated increased secretion of molecules like Vcam1, Tigit, Ifng, and Fasl to endothelial cells and fibroblasts, and a higher engagement with Nectin2 and Cxcl pathway signals, as compared to tolerance and normal conditions (Figure S6B).

### Bulk RNA-seq and Flow Cytometry Confirm the Dominance of CD103^-^CD8^+^ TRMs in Rejected Mouse Liver Transplants

**Figure 7:**
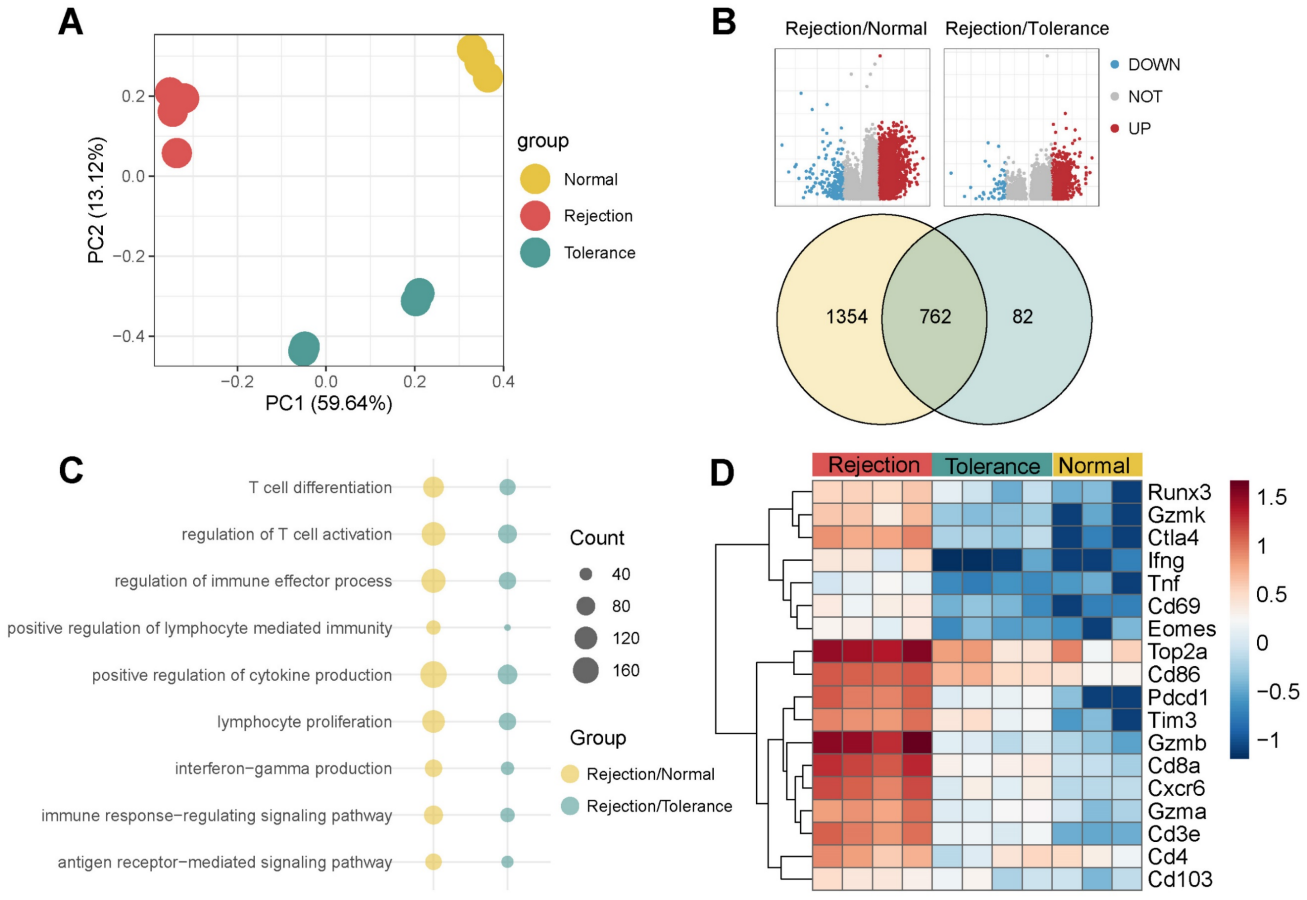
** Bulk RNA-seq analysis of Rejected Liver Transplants.** A) PCA of Bulk RNA-seq for Three Groups, Including Rejection, Tolerance, and Normal, in Mouse. B) Volcano plots and Venn plots illustrating the DEGs of rejection/normal and rejection/tolerance groups using bulk RNA-seq. C) Dot plots presenting the GO pathway enrichment analysis for DEGs of rejection/normal and rejection/tolerance groups. D) Heatmap displaying the expression of TRM-related genes among diagnoses.

Bulk RNA-seq of the three groups provided insights at the transcript level (Figure 7A). Rejection samples revealed 2,116 DEGs when compared to normal, 844 DEGs when compared to tolerance, and 762 genes common to both sets (Figure 7B). The genes identified played roles in T cell activation, proliferation, differentiation, and the regulation of cytokine and interferon-gamma (Ifng) production (Figure 7C). TRM-associated markers such as Cd8a, Runx3, Gzmb, Ctla4, Eomes, Top2a, Pd1, and Cxcr6 were expressed at higher levels in rejection than in tolerance or normal samples (Figure 7D), indicating a correlation with graft outcome.

**Figure 8:**
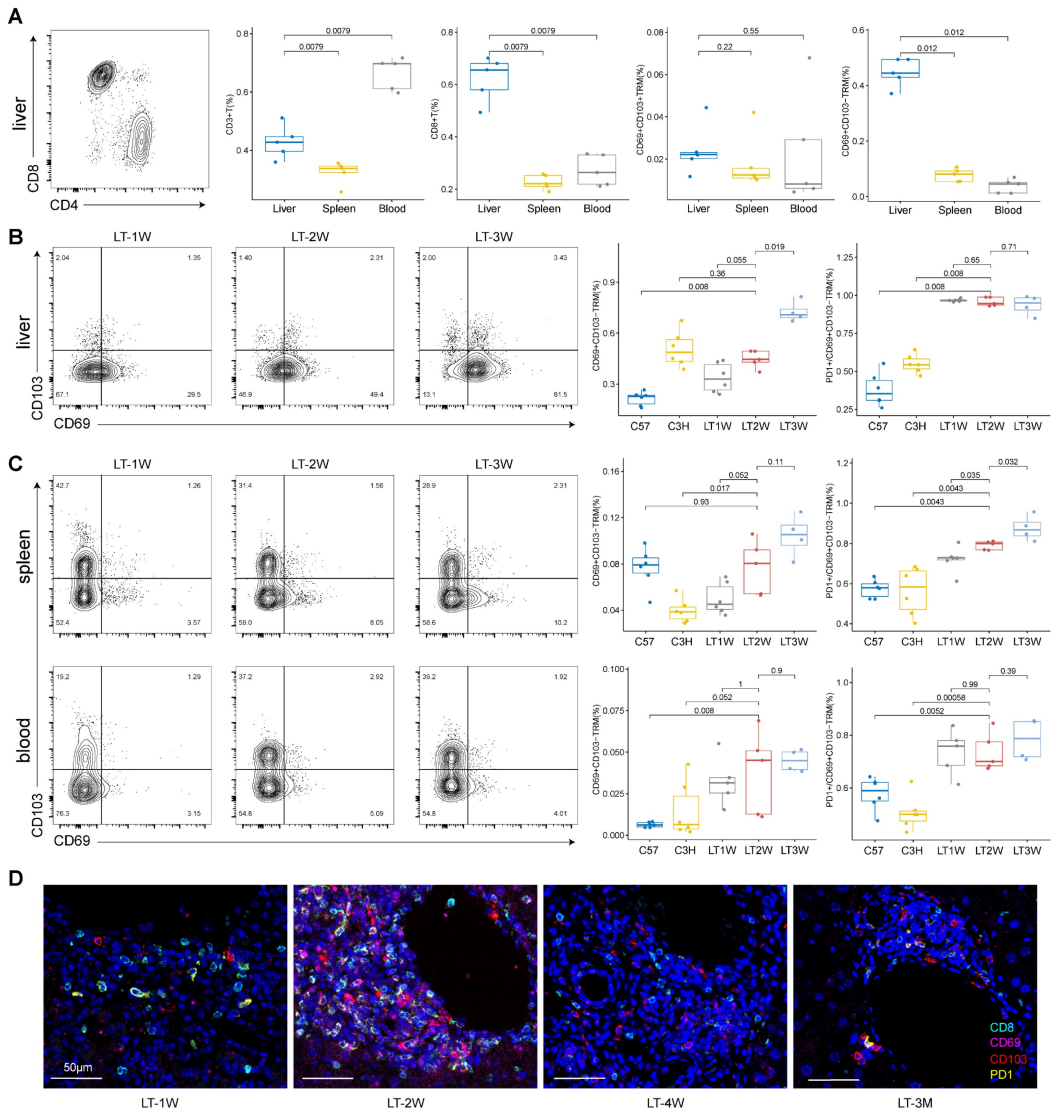
** Flow Cytometry and mIHC Confirm Dominance of CD103^-^CD8^+^ TRMs in Rejected Liver Transplants.** A) Flow cytometry depicting the distribution of CD3^+^, CD8^+^, CD69^+^CD103^+/-^ T cells among tissues (liver, spleen, and blood). B) Flow cytometry showing the dynamic changes of CD69^+^CD103^-^ and PD1^+^ CD69^+^CD103^-^ T cells in liver after different LT timelines (1W, 2W and 3W). C) Flow cytometry showing the dynamic changes of CD69^+^CD103^-^ and PD1^+^CD69^+^CD103^-^ T cells in spleen and blood after different LT timelines (1W, 2W and 3W). D) Multi-immunohistochemistry of different LT timelines (1W, 2W, 4W and 3M) in liver for CD8 (cyan), CD69 (purple), CD103 (red) and PD1 (yellow). Inset scale bar, 50 μm.

To validate the presence and prevalence of CD8^+^ TRMs within our mouse model of LT, we employed flow cytometry to examine tissues under different dynamic conditions. Our results indicated that CD3^+^ T cells were dominant in blood compared to liver and spleen, while CD8^+^ T cells showed a higher proportion in liver compared to spleen and blood. Specifically, CD103^-^CD8^+^ TRMs were more abundant than their CD103^+^ counterparts in the livers of mice with rejected transplants (Figure 8A, Figure S8).

We then compared one week (LT1W, n = 6), two weeks (LT2W, n = 5), and three weeks (LT3W, n = 4) post-transplantation in donor (C57, n = 6) and recipient (C3H, n = 6) mice. We found that increased T cell repertoire crosstalk during rejection influenced T cell subsets in the liver, spleen, and blood. CD3^+^ T cells and CD8^+^ T cells were significantly upregulated in the liver after LT (Figure S7A). CD103^-^CD8^+^ TRMs in the liver increased with the duration of transplantation, and PD1 showed higher levels within CD103^-^CD8^+^ TRMs compared to normal samples (Figure 8B).

In the spleen, CD3^+^ T cells increased after LT while CD8^+^ T cells decreased (Figure S7B). The percentage of CD103^-^CD8^+^ TRMs and PD1^+^CD103^-^CD8^+^ TRMs increased with the duration of transplantation (LT1W, LT2W, and LT3W) in the spleen (Figure 8C). In the blood, CD3^+^ T cells increased at LT2W and decreased at LT3W, while CD8^+^ T cells showed a slight increasing tendency, though not significantly (Figure S7C). Blood samples showed a low presence of CD103^-^CD8^+^ TRMs, even in LT models; however, PD1 expression was high in the limited CD103^-^CD8^+^ TRMs present (Figure 8C).

Additionally, we performed mIHC for liver samples at LT1W, LT2W, LT4W, and LT3M to show the presence and tendency of PD1^+^CD8^+^ TRMs. Samples at four weeks and three months showed a lower presence of TRMs, indicating a tolerance status (Figure 8D, Figure S7D).

## Discussion

Recent studies have underscored the significance of CD4^+^/CD8^+^ TRMs in solid organ transplantation, particularly in modulating transplant immunity 9, 11 and influencing outcomes in various liver diseases 20-22. Given the liver's distinctive immunotolerant properties, understanding the role and mechanisms of TRMs in LT is crucial 23. Through a robust multi-omics approach, our study provides an in-depth analysis of T cells in LT, emphasizing the complex phenotypic characteristics and significant roles of TRMs within the liver transplant microenvironment. We observed a pronounced dominance of CD8^+^ TRMs with distinct features in cases of liver transplant rejection, along with two distinct phenotypes of CD4^+^ TRMs. Notably, CD8^+^ TRMs exhibited dynamic levels during LT and engaged in significant signaling, particularly with Kupffer cells, involving pathways like PD-L1 and various chemokines, which are instrumental in sustaining rejection 24.

In the liver, long-lived alloreactive CD4^+^ TRMs have been documented to produce type 1 polyfunctional cytokine responses upon stimulation 22, 23, yet their specific role in transplant immunity requires further investigation. Our study explored the maintenance of CD4^+^ TRMs in both rejected and non-rejected liver transplants, noting their scarce presence in the blood. We identified two distinct subsets of CD4^+^ TRMs: AREG^+^ TRMs and IFNG^+^ TRMs. These subsets shared a set of 13 signature genes, including transcription factors such as FOSB, FOS, JUN, and NR4A1 25, 26. Notably, distinct expression patterns emerged between these subsets. AREG^+^ TRMs showed an upregulation of UBE2S, AREG, and RGR1, whereas IFNG^+^ TRMs exhibited elevated levels of GZMA, IFNG, GZMK, STAT1, STAT4, and CXCR3, suggesting a TRM1 phenotype. Interestingly, research has shown that Th17-derived AREG can promote intestinal fibrotic responses, positioning it as a potential therapeutic target for fibrosis 27. Additionally, CXCR3^+^CD4^+^ TRMs in the liver have been identified as a novel, functionally distinct, recirculating population that contributes to diverse immunosurveillance, notably marked by the expression of IFNG 22. However, our analysis did not reveal significant differences between these two CD4^+^ TRM subsets in the context of transplant diagnosis, indicating that they may not play a critical role in differentiating between rejection and non-rejection in liver transplants.

A noteworthy revelation from our research is the predominant presence of CD8^+^ TRMs in liver transplant rejection, emphasizing their distinctive transcriptional identity and dynamic nature, potentially playing a crucial role in rejection development 14. To our best knowledge, it's the first time to find the significant dominance of CD8^+^ TRMs during liver transplant rejection. However, studies found the CD8^+^ TRMs infiltration of graft using mouse kidney transplantation model, but not indicated the relation of rejection status 10, 28. We explored the plasticity of TRM cells in LT. The observed elevation of CD8^+^ TRMs during liver transplant rejection is marked by a unique expression profile. This profile encompasses an upregulation of cytotoxic markers such as GZMB and IFNG 29, 30; tissue-residency markers, including CD69, CD103, CXCR6 and CD49A 31; immune checkpoint molecules like PD1, CTLA4, and TIGIT 32 and proliferative markers PCNA and TOP2A 33. Additionally, these cells exhibit high levels of transcription factors EOMES and RUNX3, further emphasizing their distinct role in transplant rejection scenarios. Eomes was essential to molecular and functional attributes of small intestine and colon CD8^+^ TRMs 34. Runx3 was known to be important to CD8^+^ TRMs, rather than CD4^+^ TRMs 35, and previously found to promote cytotoxic function of skin CD8^+^ TRMs 13, 36. Pseudotime analysis showed TRMs were observed in the late phase, indicating a transition from effector CD8^+^ T cells, which might sustain localized rejection in the transplanted liver. Results This observation aligns with the notable conservation of certain CTRM traits across different organ transplants, highlighting the specialized immune environment of the transplanted liver.

Utilizing a mouse LT model, our study constructed a dynamic T cell atlas, with CD8^+^ TRMs, with poor expression of Cd103, exhibiting higher activity in the acute phase, especially in signaling interactions with Kupffer cells. This finding corroborates earlier reports suggesting that PD-L1 blockade can rejuvenate TRM cells, offering potential therapeutic strategies targeting the PD-L1/PD-1 pathways during LT rejection 24. CD103^-^ TRMs was found as the primary responders, reacting to secondary infection in intestinal while CD103^+^ TRMs had limited potential 37, which indicated that CD103^-^ TRMs intend to immune regulation. We also validated the dominance of CD103^-^CD8^+^ TRMs in rejection samples with high expression of immune checkpoint pathways, especially the PD-L1/PD-1 pathway, using an independent cohort. Our comprehensive use of immunological techniques including flow cytometry, immunohistochemistry, and fluorescence assays provided quantitative insights into TRM distribution and communication, enhancing our understanding of TRM behavior post-transplantation. TRMs, recognized as key mediators of adaptive immunity within nonlymphoid tissues, are characterized by their retention in these tissues without circulating in the bloodstream 38. Consistent with this, our findings indicate a notably low presence of TRMs in spleen tissues and blood, compared with liver, for LT. Furthermore, Megan Sykes found the increased blood-tissue crosstalk and dynamic changes of TRMs post intestine transplantation patients using detailed flow cytometry and omics in recent studies 39, 40, and we also investigated the blood-tissue crosstalk in LT patients.

The translational significance of our findings is considerable. The study demonstrated that T cell repertoire cross talk during rejection can influence the T cell subsets in the blood. During the period of LT with rejection, both CD3^+^ and CD8^+^ T cells increased in blood, followed by the high infiltration of CD3^+^ and CD8^+^ T cells in transplanted liver. Especially, by elucidating the functional dynamics of TRMs, especially the CD8^+^ subset, our research paves the way for novel strategies to monitor and potentially modulate the immune response in LT recipients. Targeting TRM-related pathways could offer new therapeutic avenues for enhancing graft acceptance and longevity. Additionally, the distinct behavior of TRMs in rejection scenarios presents opportunities for early detection and intervention, potentially significantly improving patient outcomes.

Limitations of our study include the lack of further basic validation through in vivo and in vitro studies to elucidate the transcription factors, cell differentiation of TRMs, and intercellular communication with other tissue-resident cells. This would deepen our understanding of TRM development and function in transplant immunity and potentially guide treatment strategies targeting TRMs.

In conclusion, this study not only enriches the current understanding of transplant immunology but also opens avenues for future research. CD8^+^ TRMs have a potential function of immune regulation in LT, which exhibit an activated state, secret cytotoxic factors and communicate with other tissue resident cells, especially for rejection. The therapeutic manipulation of TRM responses to foster tolerance and reduce rejection risks, coupled with a more nuanced approach to immunosuppressive therapy informed by specific immune cell populations, could revolutionize LT management and outcomes.

## Materials and Methods

### Study Subjects

This study encompassed patients who underwent liver transplantation (LT) at the Organ Transplantation Center, the Affiliated Hospital of Qingdao University, between November 2020 and November 2023. Liver biopsies, including a previously published cohort 16, were conducted post-LT. All liver grafts originated from voluntary donations after cardiac death or living donors. The Ethics Committee of the Affiliated Hospital of Qingdao University (IRB number: QYFYWZLL26550) granted approval for the study.

### Animals

Male C57BL/6J mice and male C3H/He mice served as donors, with male C3H/He mice as recipients. All animals, aged 8-10 weeks (Body Weight = 23 ± 2 g), were procured from SiPeiFu, Beijing, Biotechnology Co., LTD, and were housed in a specific pathogen-free (SPF) environment. Orthotopic liver transplantation (OLT) surgeries were performed under isoflurane inhalation anesthesia, following established procedures 41.

### Tissue Dissection and Cell Suspension

Human liver biopsies and mouse liver tissues, obtained immediately post-surgery, were immersed in a tissue preservation solution and transported for standard dissociation procedures. The tissues were finely diced into approximately 0.5-mm³ pieces in RPMI-1640 medium (Invitrogen) supplemented with 1% Penicillin/Streptomycin. Enzymatic digestion was performed using a mix containing 0.05% trypsin (INVITROGEN Cat# 25200056), 0.4% collagenase IV (INVITROGEN Cat# 17104-019), 0.25% collagenase I (SIGMA Cat# C0130-1G), 0.13% collagenase II (BBI Cat# A004174-0001), and 0.1% elastinase (WORTHINGTON Cat# LS002292), maintained at 37°C for 30-45 minutes with constant agitation. Following digestion, the cell suspensions were filtered through 70-μm and 40-μm cell strainers (BD) and centrifuged at 300g for 10 minutes. The supernatant was discarded, and the cell pellet was resuspended in red blood cell lysis buffer (Thermo Fisher), incubated on ice for 3 minutes to lyse any remaining red blood cells. After two washes with PBS (Invitrogen), the cells were finally resuspended in PBS containing 0.04% BSA for further analysis.

### Library Preparation and Sequencing

For scRNA-seq library preparation, the DNBelab C Series High-throughput Single-cell System (BGI-research) was utilised. This involved transforming single-cell suspensions into barcoded scRNA-seq libraries, encompassing droplet encapsulation, emulsion disruption, collection of mRNA-captured beads, reverse transcription, and subsequent cDNA amplification and purification. The resulting cDNA was fragmented into segments ranging from 250 to 400 base pairs. Indexed sequencing libraries were then constructed following the manufacturer's guidelines. Quality control checks were performed using the Qubit ssDNA Assay Kit (Thermo Fisher Scientific) and the Agilent Bioanalyzer 2100. Sequencing was conducted on the DIPSEQ T1 platform (China National GeneBank), utilizing paired-end sequencing. The sequencing read structure comprised a 30-base pair (bp) Read 1, including a 10-bp cell barcode 1, a 10-bp cell barcode 2, and a 10-bp unique molecular identifier (UMI). This was followed by a 100-bp Read 2 for gene sequencing and a 10-bp barcode read for sample indexing.

### Single-Cell RNA Sequencing Data Pre-Processing

The sequencing data underwent comprehensive processing using an open-source pipeline available on GitHub (https://github.com/MGI-tech-bioinformatics/DNBelab_C_Series_HT_scRNA-analysis-software). The process initiated with sample de-multiplexing, barcode processing, and the counting of single-cell 3' unique molecular identifiers (UMIs) using the default parameters of the pipeline. The resulting reads were aligned to the GRCh38 human genome reference using STAR software (version 2.5.3). Cell validity was ascertained based on UMI distribution per cell, employing the barcodeRanks() function from the DropletUtils tool. This facilitated the removal of background noise and beads with UMI counts below a specified threshold. For the final step, we employed the PISA framework to quantify gene expression in cells, generating a gene-by-cell matrix for each library.

### Single-Cell Gene Expression Quantification and Subcluster Delineation

The analysis of our single-cell RNA sequencing data utilized the Seurat R package (version 4.3.0) 42 to manage various stages of data processing. This began with raw data importation, followed by stringent quality control measures. Low-quality cells, defined as those with fewer than 501 expressed genes and exhibiting more than 25% mitochondrial counts, were filtered out. High-quality cells passing these filters were then normalized and scaled using Seurat's default settings.

Next, we identified highly variable features within our dataset using the FindVariableFeatures function. This step was crucial for the subsequent principal component analysis (PCA) on the scaled data, focusing on these variable features. We proceeded with dimension reduction and clustering of the data, employing the FindNeighbors (with dimensions set to 1:10) and FindClusters (resolution set at 0.5) functions within Seurat. The final step in our analytical process was the application of uniform manifold approximation and projection (UMAP). This technique was instrumental in further exploring and visualizing intricate patterns within our dataset.

### Cell Type Determination

To identify differentially expressed features across each cell cluster, we utilized the FindMarkers and FindAllMarkers functions. Cell types were annotated based on known biological types, using a set of canonical marker genes (Table S1). This annotation process was guided by the CellMarker database (http://bio-bigdata.hrbmu.edu.cn/CellMarker/) and corroborated by information from published articles. Additionally, for more robust cell type identification, we employed the SingleR package (version 2.0.0) 43. This package further aided in the accurate determination of cell types by aligning our single-cell RNA sequencing data with reference datasets.

### Functional Enrichment Analysis and TRM Scores

Upon completing the annotation of each cell type, we undertook a functional enrichment analysis of differentially expressed genes across various cell clusters. This analysis, essential for elucidating the biological processes and potential functions of distinct cell types, employed Gene Ontology (GO) and KEGG (Kyoto Encyclopedia of Genes and Genomes) pathways. To conduct this analysis, we utilized the clusterProfiler package (version 3.17.0) 44 along with the org.Hs.eg.db package (version 3.11.4). The p-value cutoffs for both GO and KEGG analyses were set at 0.05 to ensure the significance of our findings. The top ten terms derived from these results were visually represented in the form of barplots or dotplots, offering a clear and concise graphical representation of the key functional attributes associated with each cell type.

For TRM scores with gene set analysis, we utilized the 'AddModuleScore' function within the Seurat package, based on the TRM related markers 45. This approach involved the application of specific gene sets of interest, which were sourced from previously published studies or datasets. We then computed a score for each cell, derived from the expression levels of genes within each gene set, thereby enabling a detailed cell-by-cell analysis of gene expression patterns.

### Pseudotime Analysis

We performed trajectory analysis using the Monocle package (version 2.28.0) 46, allowing us to track the developmental progression of cells in a pseudo-temporal order. This analysis encompassed various T subsets, including TRM, TEX, and naïve T cells. Specific parameters were set for each cell group: a lower detection limit of 0.5, a minimum expression threshold of 0.1, and a requirement for the gene to be expressed in at least 10 cells. Visualization of cell trajectories was achieved using the plot_cell_trajectory function, mapping out potential developmental pathways of cells based on pseudotime, Seurat cluster assignments, and additional metadata. This provided a dynamic view of cellular differentiation and maturation processes.

### Cell-Cell Communication Analysis

In our investigation into intercellular interactions, we employed the CellChat package (version 1.6.1) 47, focusing on ligand-receptor interactions based on the KEGG signaling pathway database and recent experimental studies. The analysis comprised several crucial steps, starting with the identification of differentially expressed signaling genes to highlight those with significant expression variations. This was followed by calculating the ensemble average expression, providing a comprehensive view of gene expression patterns across different cell types. The final step involved assessing the probability of intercellular communication, pivotal in understanding the complex signaling networks and interactions among various cell populations in our study.

### Bulk RNA Sequencing Analysis

To validate our findings, we sourced transcriptome data from the NCBI Gene Expression Omnibus (GEO). The specific datasets accessed were GSE145780 48 and GSE203453 49, encompassing gene expression profiles obtained from microarrays of liver transplant biopsies. These biopsies included samples from both rejection and non-rejection scenarios, providing a comprehensive view of gene expression alterations associated with these different transplant outcomes.

### Immune Infiltration Estimation

To delineate the intricate immune cell infiltration within tissue samples, we employed the CIBERSORT algorithm 50. Developed by Newman et al. from the Alizadeh Lab, this method is esteemed for its efficacy in decoding complex immune landscapes based on gene expression profiles. CIBERSORT's robust computational approach allows for a detailed and accurate estimation of the immune cell composition within our tissue samples, providing valuable insights into the immunological milieu of LT.

### Histological Staining

Fresh liver tissue samples were initially fixed in 4% paraformaldehyde and subsequently embedded in paraffin. These samples were then sectioned into 4 μm thick slices. For morphological assessment, the sections underwent staining using hematoxylin and eosin (H&E). Additionally, reticular fiber staining was performed to evaluate tissue architecture, and Masson's trichrome staining was applied to specifically detect and highlight areas of fibrosis within the liver tissues.

### Immunohistochemical (IHC) Staining Analysis

IHC staining was conducted on liver tissue sections to target specific antigens, including CD3, CD4, CD8A, CD69, CD103, and PD1. Initially, the tissue slides underwent deparaffinization and dehydration. Following this, they were immersed in Tris-EDTA antigen retrieval buffer at pH 6.0 and pH 9.0 and subjected to heat-induced antigen retrieval. To neutralize endogenous peroxidases and block nonspecific antigens, 3% hydrogen peroxide (H2O2) and 3% bovine serum albumin (BSA) were applied, respectively. Subsequent to these preparatory steps, the slides were sequentially incubated with primary antibodies: anti-CD3, anti-CD4, anti-CD8A, anti-CD69, anti-CD103, and anti-PD1. For signal detection, a TSA kit from Nanjing Freethinking Biotechnology Co., Ltd. (China) was utilized. Nuclei counterstaining was performed using DAPI. Finally, the stained slides were scanned using a Pannoramic MIDI slice scanner (3Dhistech, Hungary), and image analysis was carried out employing the HALO 2.0 Area Quantification algorithm (Indica Labs; Corrales, NM) at Nanjing Freethinking Biotechnology Co., Ltd. (China).

### Flow Cytometry

For flow cytometric analysis, cells were stained with a panel of fluorochrome-conjugated antibodies. This panel included anti-CD3, CD4, CD8A, CD69, CD103 and PD1, all sourced from BioLegend. Staining procedures were carried out in strict accordance with the manufacturer's protocols. Following staining, the samples were subjected to analysis using FlowJo software (verision 10.8.1), enabling detailed examination and quantification of various cell populations based on their fluorescent properties and antibody binding characteristics.

### Statistical analysis

Statistical analysis and graphical representation of the data were performed with R software (version 4.2.3). The data are presented as mean ± standard error of the mean (SEM). An unpaired student t test was used to determine the statistical significance between two groups. Statistical significance was indicated as *P < 0.05, ** P < 0.01, *** P < 0.001.

## Supplementary Material

Supplementary figures.

Supplementary table 1 meta data.

Supplementary table 2 human CD4T.

Supplementary table 3 human CD8T.

Supplementary table 4 GO KEGG.
