# Supplementary material for: Single cell RNA-sequencing delineates CD8+ tissue resident memory T cells maintaining rejection in liver transplantation
Source: Theranostics. 2024 Aug 12;14(12):4844–60. doi: 10.7150/thno.96928 (PMC11373625; doi:10.7150/thno.96928)
Supplement: Supplementary file 1 — Supplementary figures. [file thnov14p4844s1.pdf]

Supplementary figures and figure legends

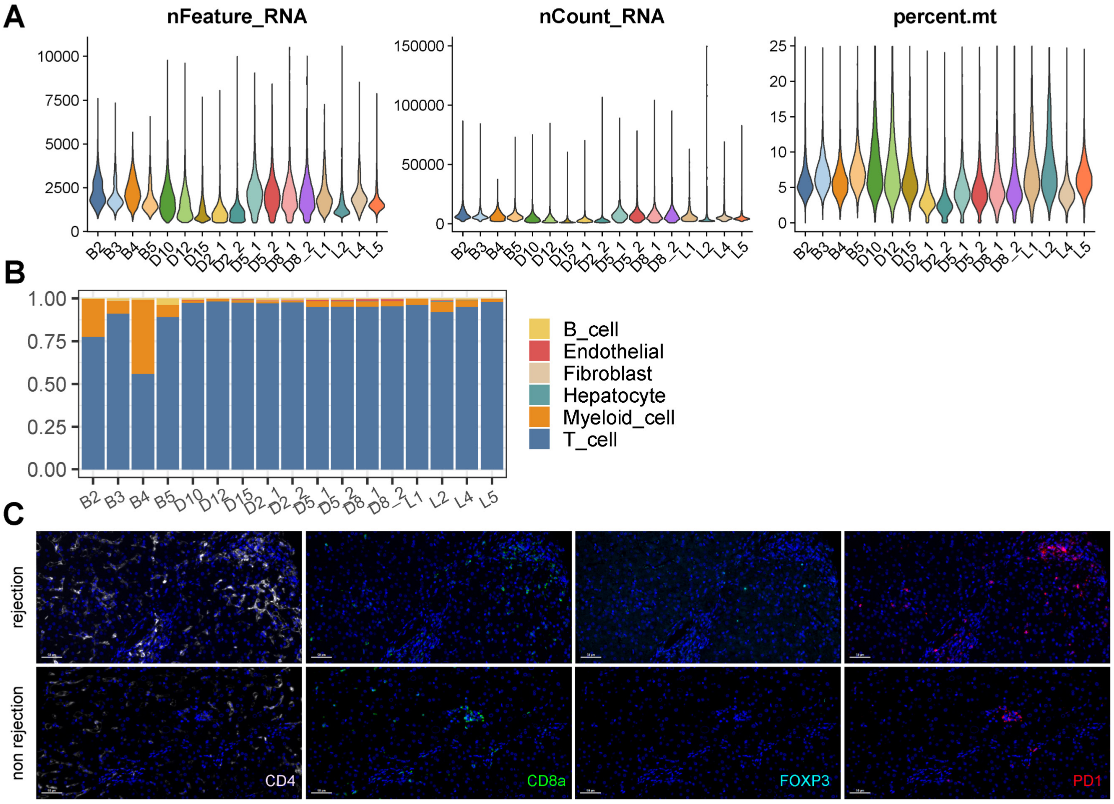

Figure S1. Single-cell Atlas of Human Liver Transplantation. A) Violin plots displaying nFeature_RNA, nCount_RNA, and percent.mt (referring to the number of genes, UMIs, and the percentage of mitochondrial genes, respectively) used for quality control among samples. B) Bar plots presenting the proportion of cell types in each sample. C) Multi-immunohistochemistry of rejection and non-rejection transplanted liver for CD4 (white), CD8 (green), FOXP3 (cyan), and PD1 (red). Whole image scale bar, 200 μm. Inset scale bar, 50 μm.

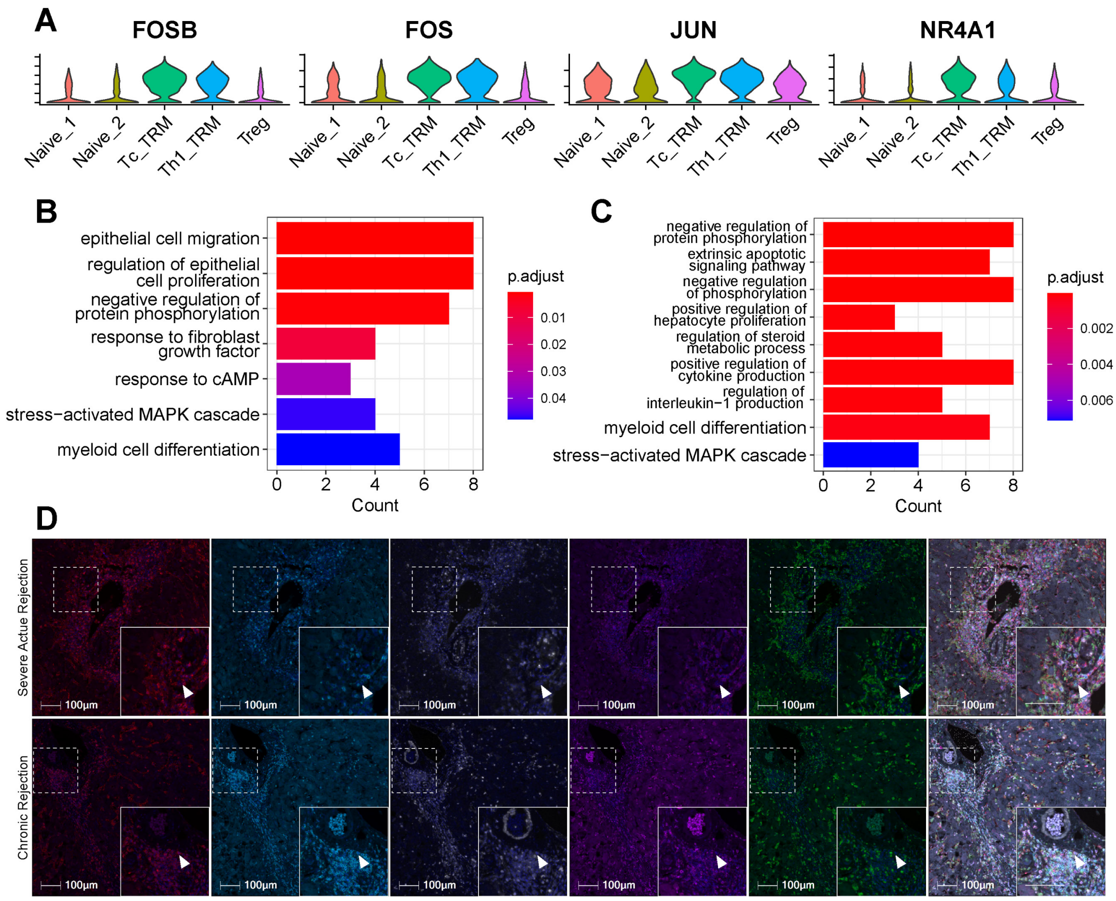

Figure S2. A) Violin plots showing the expression of FOSB, FOS, JUN and NR4A1 across $CD4^+$ T subsets. B) Bar Plots Displaying the GO Pathway Enrichment Analysis for DEGs of $CD4^+$ TRMs. C) Bar Plots Displaying the KEGG Pathway Enrichment Analysis for DEGs of $CD4^+$ TRMs. D) Multi-immunohistochemistry of rejection-transplanted liver for DAPI (blue), CD4 (red), CD69 (cyan), CD103 (white), PD1 (purple), and CD68 (green). White arrows point to cells co-expressing CD4, CD69, and CD103. Whole image/insert scale bar, 100 μm.

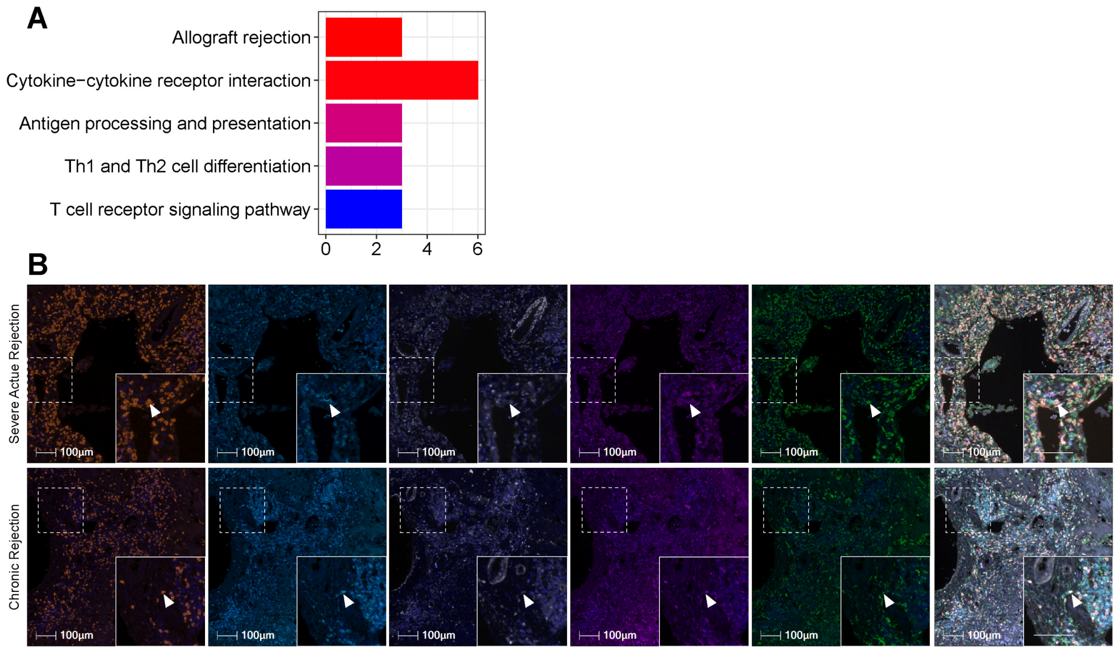

Figure S3. A) Bar Plots Displaying the KEGG Pathway Enrichment Analysis for DEGs of CD8^{+} TRMs. B) Multi-immunohistochemistry of rejection-transplanted liver for DAPI (blue), CD8 (orange), CD69 (cyan), CD103 (white), PD1 (purple), and CD68 (green). White arrows point to cells co-expressing CD8, CD69, CD103, and PD1. Whole image/insert scale bar, 100 µm.

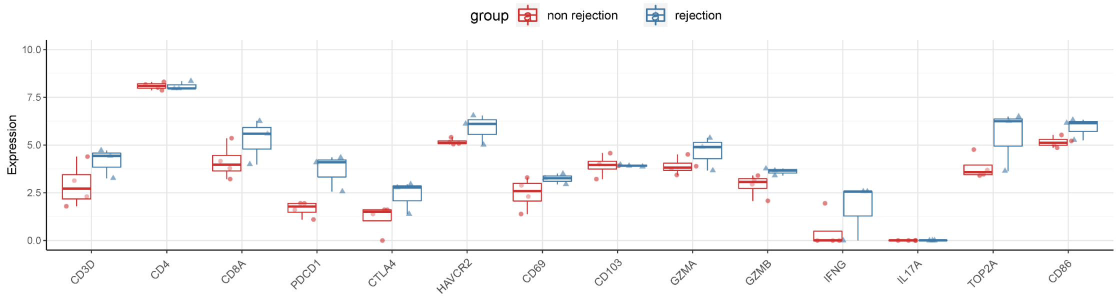

Figure S4. Gene Expression of TRM-related Markers between Non-rejection (n = 3) and Rejection (n = 4) Samples Using Bulk RNA-seq with Another Cohort.

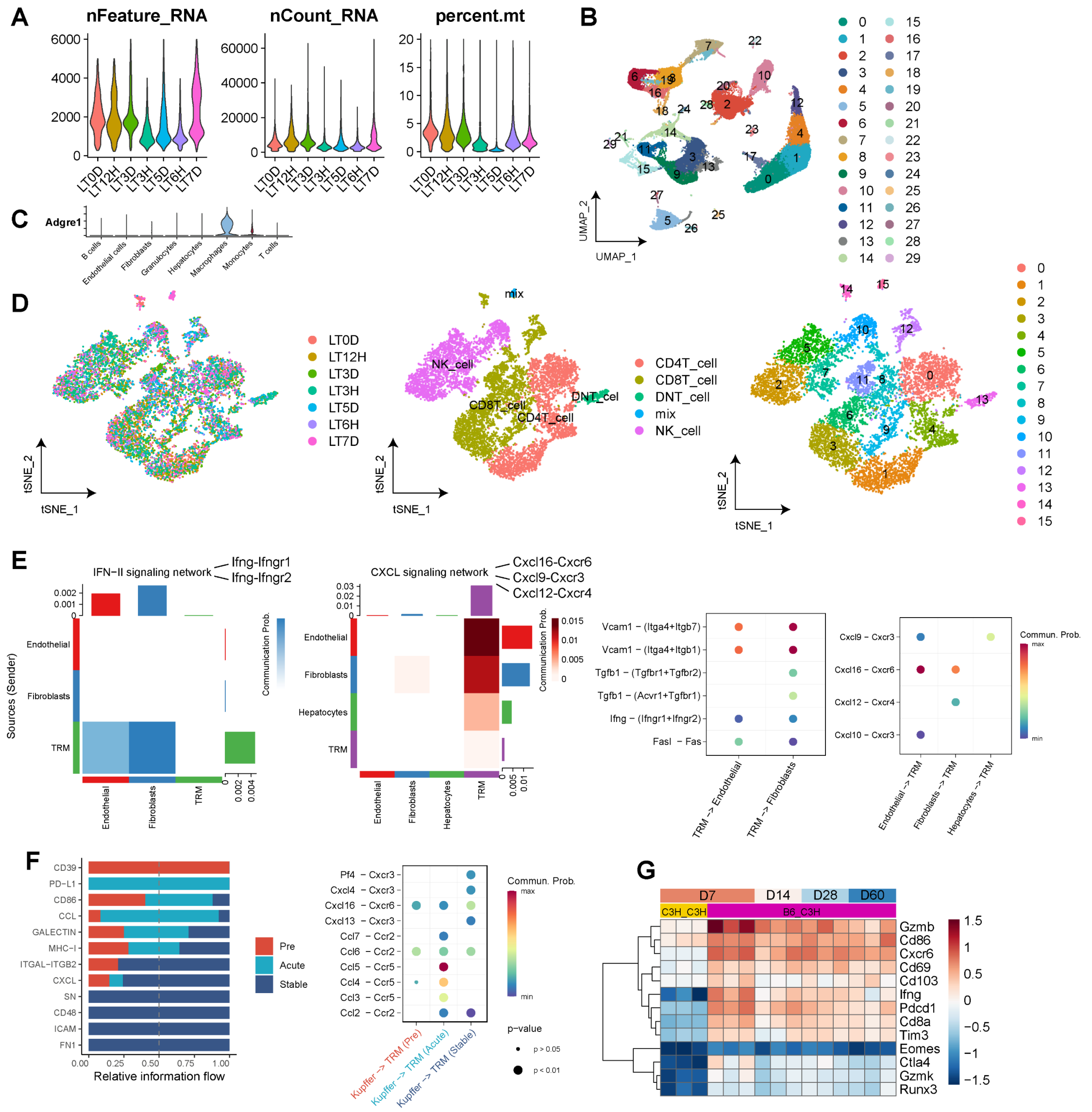

Figure S5. Single-cell Analysis for Dynamic Timeline Phenotypes in Mouse Liver Transplantation Models. A) Violin plots displaying nFeature_RNA, nCount_RNA, and percent.mt used for quality control among samples. B) UMAP plot for clustering all cells in mouse LT with 30 clusters. C) Expression of Myeloid cell marker Adgre1. D) tSNE plots showing the dynamic timelines, annotations, and re-clustering for all T and NK cells. E) Heatmaps and dot plots showing the cellular communication between CD8$^+$ TRMs and stromal cells, including endothelial cell, fibroblast, and hepatocyte. F) Bar plots and dot plots illustrating the cellular communication between CD8$^+$ TRMs and Kupffer cells. Red indicating Pre, Cyan indicating Acute, and blue indicating Stable. G) Heatmap showing the expression of TRM-related genes among different timelines (7d, 14d, 28d, and 60d) using bulk RNA-seq.

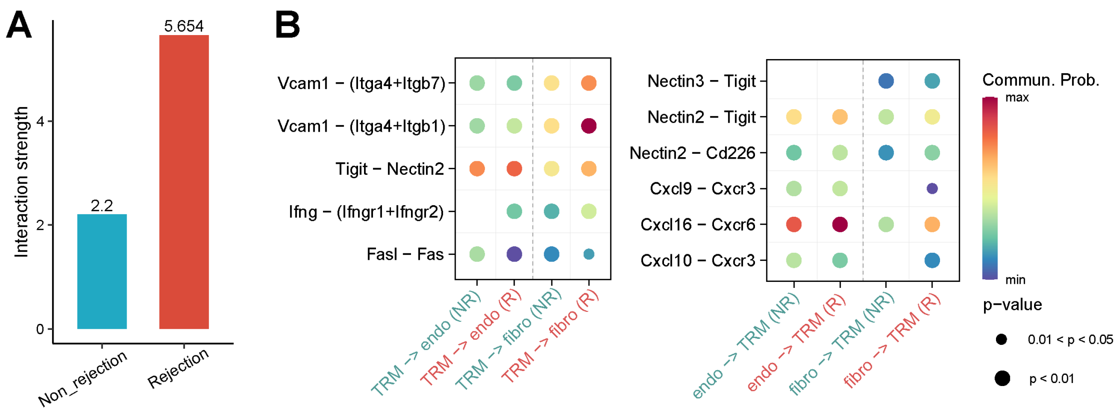

Figure S6. Bar Plots (A) and Dot Plots (B) Displaying the Cellular Communication Between CD8^{+} TRMs and Stromal Cells, Including Endothelial Cell and Fibroblast. Red indicating rejection and cyan indicating non-rejection.

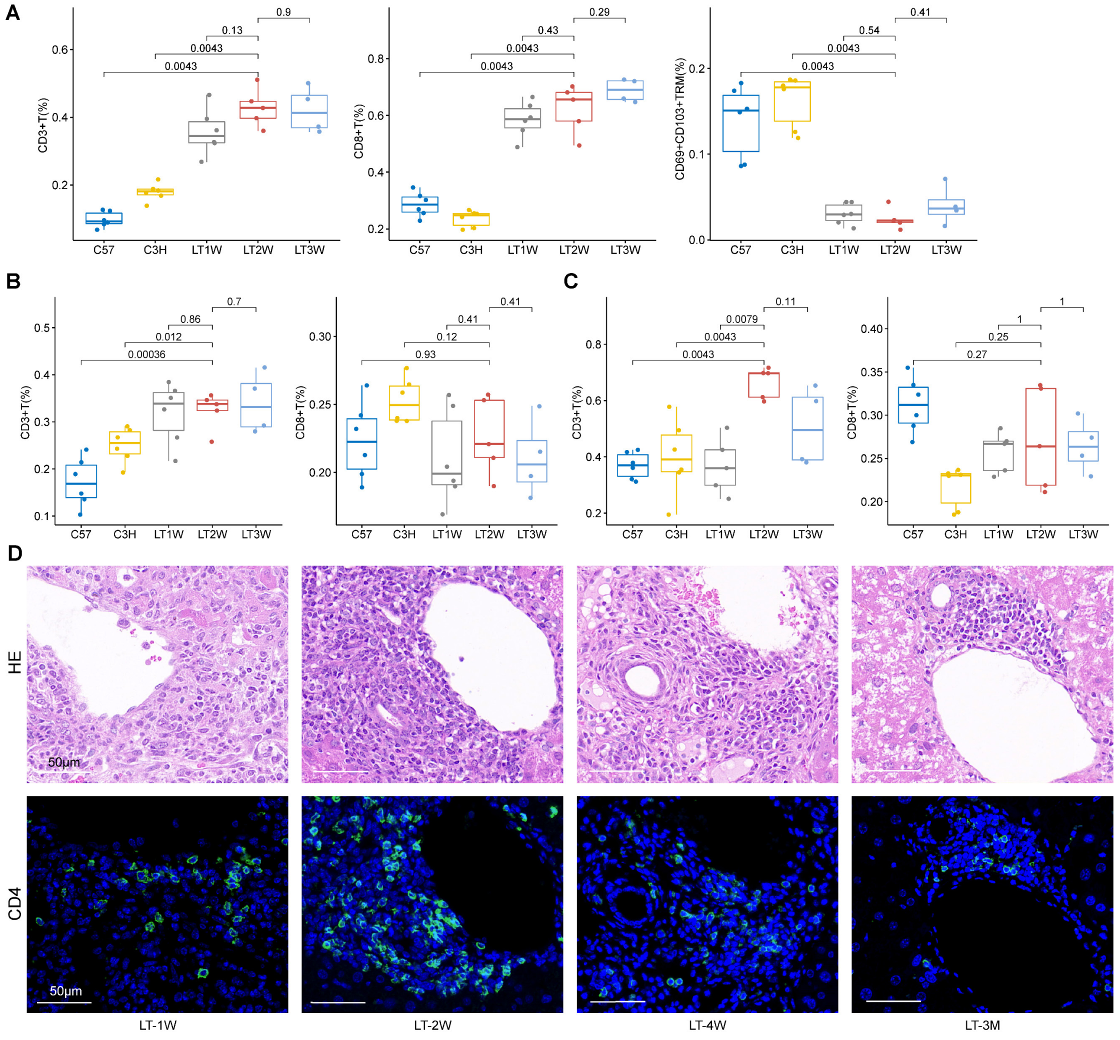

Figure S7. Flow Cytometry and mIHC Confirm Dominance of CD103^{-}CD8^{+} TRMs in Rejected Liver Transplants. A) Flow cytometry showing the dynamic changes of CD3^{+}, CD8^{+} and CD69^{+}CD103^{+} T cells in liver after different LT timelines (1W, 2W and 3W). B-C) Flow cytometry showing the dynamic changes of CD3^{+}, CD8^{+} and CD69^{+}CD103^{+} T cells in spleen (B) and blood (C) after different LT timelines (1W, 2W

and 3W). D) HE and multi-immunohistochemistry of different LT timelines (1W, 2W, 4W and 3M) in liver for CD4 (green). Inset scale bar, 50 μm.

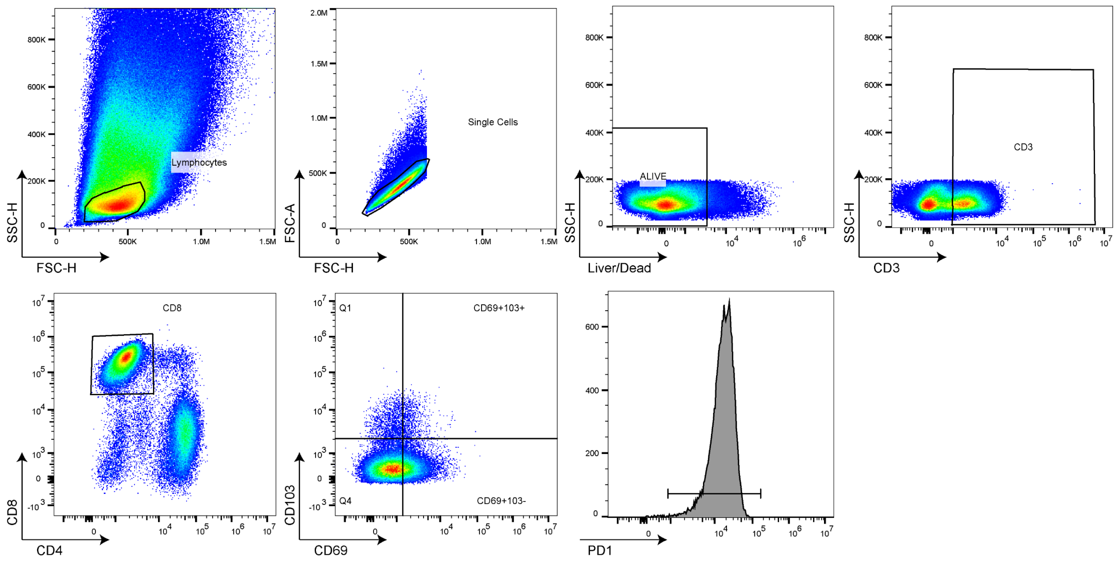

Figure S8. Gating strategy to identify CD8^{+}CD69^{+}CD103$^{+/-}$ T cells.
